# Activation of complement factor B contributes to murine and human myocardial ischemia/reperfusion injury

**DOI:** 10.1371/journal.pone.0179450

**Published:** 2017-06-29

**Authors:** Nicholas Chun, Ala S. Haddadin, Junying Liu, Yunfang Hou, Karen A. Wong, Daniel Lee, Julie I. Rushbrook, Karan Gulaya, Roberta Hines, Tamika Hollis, Beatriz Nistal Nuno, Abeel A. Mangi, Sabet Hashim, Marcela Pekna, Amy Catalfamo, Hsiao-ying Chin, Foramben Patel, Sravani Rayala, Ketan Shevde, Peter S. Heeger, Ming Zhang

**Affiliations:** 1 Nephrology Division, Department of Medicine and Translational Transplant Research Center, Icahn School of Medicine at Mount Sinai, New York, New York, United States of America; 2 Department of Anesthesiology, Yale University School of Medicine, New Haven, Connecticut, United States of America; 3 Department of Anesthesiology, College of Medicine, SUNY Downstate Medical Center, Brooklyn, New York, United States of America; 4 Department of Surgery, College of Medicine, SUNY Downstate Medical Center, Brooklyn, New York, United States of America; 5 Department of Surgery, Yale University School of Medicine, New Haven, Connecticut, United States of America; 6 Department of Medical Chemistry and Cell Biology, Göteborg University, Göteborg, Sweden; 7 Department of Biomedical Sciences, Long Island University, Brookville, New York, United States of America; 8 Department of Cell Biology, College of Medicine, SUNY Downstate Medical Center, Brooklyn, New York, United States of America; Macau University of Science and Technology, MACAO

## Abstract

The pathophysiology of myocardial injury that results from cardiac ischemia and reperfusion (I/R) is incompletely understood. Experimental evidence from murine models indicates that innate immune mechanisms including complement activation via the classical and lectin pathways are crucial. Whether factor B (fB), a component of the alternative complement pathway required for amplification of complement cascade activation, participates in the pathophysiology of myocardial I/R injury has not been addressed. We induced regional myocardial I/R injury by transient coronary ligation in WT C57BL/6 mice, a manipulation that resulted in marked myocardial necrosis associated with activation of fB protein and myocardial deposition of C3 activation products. In contrast, in fB^-/-^ mice, the same procedure resulted in significantly reduced myocardial necrosis (% ventricular tissue necrotic; fB^-/-^ mice, 20 ± 4%; WT mice, 45 ± 3%; *P* < 0.05) and diminished deposition of C3 activation products in the myocardial tissue (fB^-/-^ mice, 0 ± 0%; WT mice, 31 ± 6%; *P*<0.05). Reconstitution of fB^-/-^ mice with WT serum followed by cardiac I/R restored the myocardial necrosis and activated C3 deposition in the myocardium. In translational human studies we measured levels of activated fB (Bb) in intracoronary blood samples obtained during cardio-pulmonary bypass surgery before and after aortic cross clamping (AXCL), during which global heart ischemia was induced. Intracoronary Bb increased immediately after AXCL, and the levels were directly correlated with peripheral blood levels of cardiac troponin I, an established biomarker of myocardial necrosis (Spearman coefficient = 0.465, *P* < 0.01). Taken together, our results support the conclusion that circulating fB is a crucial pathophysiological amplifier of I/R-induced, complement-dependent myocardial necrosis and identify fB as a potential therapeutic target for prevention of human myocardial I/R injury.

## Introduction

Myocardial ischemia from inadequate coronary perfusion can occur regionally (e.g. due to coronary atherosclerosis) or globally (e.g. following aortic cross clamping (AXCL) during bypass surgery) and leads to myocardial necrosis. Prolonged regional myocardial ischemia manifests clinically as myocardial infarction (MI) while global cardiac ischemia is commonly subclinical and manifests as an increase in the peripheral blood of cardiac troponin I (cTnI) [1–9]. Emerging evidence suggests that small post-surgical elevations in cTnI can negatively impact long-term outcomes [10, 11], underscoring the importance of such subclinical injury. Reperfusion of the ischemic heart tissue with thrombolytic therapy, percutaneous coronary intervention, or following release of aortic cross clamping can acutely limit the necrotic damage but paradoxically may elicit an inflammatory response that contributes to further tissue damage. The induced reperfusion injury can stun the myocardium (limiting contractile function) and induce non-necrotic forms of cell death [12–15]. Understanding the molecular mechanisms underlying myocardial I/R injury is vital for future design of therapeutic interventions aimed at improving survival and reducing long term morbidity.

The discovery of a protective effect of “ischemic pre-conditioning” in a canine cardiac I/R model by Murry et al. offered hope for nonpharmacological interventions in I/R injury [16]. Since then, various cardioprotective strategies have been proposed to condition the heart directly or indirectly through brief episodes of ischemia and reperfusion. These include ischemic preconditioning, ischemic post-conditioning and remote ischemic conditioning, all of which showed success in animal models and small clinical studies (reviewed in-depth elsewhere) [17–22]. However, two recent large clinical trials investigating the role of remote ischemic preconditioning in cardiac surgery (RIPHeart [23] and ERICCA [24]) had negative outcomes.

Similarly, a number of pharmacological interventions to reduce myocardial damage have also been studied in clinical trials without encouraging results. One recent trial with the CypD inhibitor Cyclosporine-A (the CIRCUS Trial) showed no benefit in long term clinical outcome [25] and while β-blockers greatly improve overall clinical outcomes [26–28], their effect on infarct size in STEMI patients is still debatable [18]. There are other promising cardioprotective agents yet to be evaluated in clinical trials, e.g. glucose modulators, cyclooxygenase (COX)- and lipoxygenase (LOX)-directed lipid mediators [29, 30] and Exendin-4 [31], but the current absence of effective cardioprotective therapies supports the need for identifying novel therapeutic targets potentially capable of limiting cardiac ischemic injury

Current concepts of mechanisms leading to I/R injury [18, 32–35] are that after reperfusion, activation of a number of intracellular signaling pathways leads to calcium influx [36–39], mitochondrial dysfunction [40–45], production of reactive oxygen species [46–49], and activation of proteases [50]. These intracellular changes can directly cause cell death and can activate the vascular endothelium to express adhesion molecules, release proinflammatory cytokines and chemokines, and upregulate production of complement components [51–55]. Complement activation synergizes with toll-like receptor (TLR) signals induced by ligation of damage-associated molecular patterns (DAMPs), including HMGB1, [56–60] to upregulate NF-κB [61, 62] and amplifies local inflammation [63–65] and recruitment of inflammatory cells [66–68].

Complement activation can be initiated by the classical, mannose binding lectin (MBL), and alternative pathways that converge at the generation of C3-convertases. A common central factor B (fB)-dependent amplification loop continually generates C3-convertases, resulting in production of the potent yet short-lived anaphylatoxin C3a and the opsonin C3b, and has been shown to be critical in pathogen-induced inflammation [69, 70]. Subsequent common terminal pathway (C5-9) activation generates another anaphylatoxin, C5a, and causes formation and deposition of the membrane-attack complex (MAC) on cell surfaces [71–75] which can lead to NF-kB-dependent inflammatory responses [69, 70, 76] While prior work by our group among others focused on the role of the classical and MBL pathways as initiators of complement-dependent inflammation in myocardial I/R injury, [69–75, 77–81] the role of fB, required for amplification of the complement cascade, has not been clearly delineated. To test the hypothesis that fB is a key mediator of complement-dependent myocardial I/R injury, we studied surgically-induced cardiac I/R using fB^-/-^ mice and serum samples obtained from patients undergoing global heart ischemia during cardiac surgery. Together our new translational data provide evidence that fB is a key mediator of myocardial I/R injury.

## Materials and methods

### Mouse model of surgically-induced myocardial I/R injury

Complement fB knockout (fB^-/-^) mice and wild type (WT) littermates were generated by co-author Dr. Marcella Pekna [82] and maintained at the SUNY Downstate Medical Center Department of Laboratory Animal Resources. Genotyping was provided by GeneTyper (New York, NY) using established PCR protocols [82]. Male mice were used at 10–12 weeks of age (weights 26-30g) in accordance with the requirements of the NIH and the Institutional Animal Care and Use Committee (IACUC) of SUNY Downstate Medical Center. The protocol was approved by the IACUC of SUNY Downstate Medical Center (Approval#11–10276).

We used an established model of myocardial I/R injury model [77, 83] in which mice were anesthetized using pentobarbital sodium (60 mg/kg, i.p.), intubated and ventilated with a mouse ventilator (Harvard Apparatus, MA). After midline sternotomy, the left anterior descending artery (LAD) was ligated for 1 hour; occlusion of the LAD was confirmed by the color change of myocardial tissue and the ST elevation on ECG. Reperfusion was established and verified by the color change of the left ventricle and the appropriate ECG changes. Postoperative management included fluid replacement with normal saline and pain relief with the analgesic buprenorphine (0.1 mg/kg, intramuscularly). The mice were sacrificed after 24h (euthanized in CO_2_ chamber at Downstate facility), serum was collected and the hearts were harvested for histopathology analyses.

### Evaluation of murine myocardial necrosis by fluorescence using two probes delivered *in vivo*

A fluorescent method for tracking necrosis (initially developed by others [84, 85] and further refined by us [83]) was used. Shortly before the end of the reperfusion period described above in Materials and Methods, and before tissue harvesting, mice were anesthetized, intubated as described above, and injected i.v. with propidium iodide (PI), which enters damaged cells, intercalates with DNA and fluoresces, thus identifying necrotic tissue. The LAD was then re-occluded before heart harvesting and blue fluorescent microspheres (BFM, ThermoFisher, PA) were injected through the aortic arch to delineate the non-ischemic region of the heart. The heart was harvested and atrium was removed. The ventricle was sectioned into four slices (~1mm thickness), which were weighed and imaged under a fluorescent microscope (Olympus, PA), using the red fluorescent channel for PI, the blue for BFM.

The percentage of the tissue in a heart which was at risk for necrosis (no blue fluorescence) and which became necrotic (had red fluorescence) was determined by computerized planimetry (Image J, MD) and by the following equations:

$$\underline{\text{Weight of necrotic tissue}}=\;\left({\text{A}}_{1}{\text{ x Wt}}_{1}\right)+\left({\text{A}}_{2}{\text{ x Wt}}_{2}\right)+\left({\text{A}}_{3}{\text{ x Wt}}_{3}\right)+\left({\text{A}}_{4}{\text{ x Wt}}_{4}\right)\text{,}$$

where A was the percentage of the area of a slice staining for necrosis (red fluorescence) measured by planimetry (average of both sides of a slice) and Wt was the weight of that slice of ventricle.

$$\underline{\text{Weight of tissue at risk for necrosis}}\;(\underline{\text{w}}\text{eight }\underline{\text{a}}\text{t }\underline{\text{r}}\text{isk, WAR})=\left({\text{R}}_{1}{\text{ x Wt}}_{1}\right)+\left({\text{R}}_{2}{\text{ x Wt}}_{2}\right)+\left({\text{R}}_{3}{\text{ x Wt}}_{3}\right)+\left({\text{R}}_{4}{\text{ x Wt}}_{4}\right)\text{,}$$

where R is the percentage of the area of a slice which lacked the blue fluorescence of BFM, determined by planimetry (average of both sides of a slice used). In all cases, the tissue with red fluorescence was within the boundary of the tissue which lacked blue fluorescence.

Percentage of the weight of a ventricle at risk for necrosis which became necrotic = (weight of necrotic tissue / WAR) x 100.

Power analyses performed using G*Power 3.1 [86] showed >99% power for detection of differences in infarct size with 6–8 animals per group.

### Western blotting of murine and patients’ fB and its active fragment, Bb

Following SDS polyacrylamide gel electrophoresis, proteins were transferred to a nitrocellulose membrane. A constant amount of plasma from a healthy individual was run on each gel of human samples to control for inter-gel differences in band staining. The membranes were blocked with bovine serum albumin in 20 mM Tris, 0.9% NaCl and 2% Tween-20 and incubated with polyclonal goat anti-fB antibody (Complement Technology, TX) which detects the fB, Ba and Bb proteins separated by electrophoresis. After washing, the membrane with murine samples was incubated with a donkey anti-goat antibody conjugated with AP (Rockland, PA) and developed with BCIP/NBT Substrate System (KPL, MD). The membranes with human samples were incubated with rabbit anti-goat IgG conjugated with alkaline phosphatase (Abcam, MA) and developed with the BCIP/NBT Substrate System. Quantification of bands of interest was carried out using the ImageJ program (NIH). The intensities of bands in the human samples were normalized to that of the 93 kDa fB band of the normal control lane and expressed as relative intensity.

### Quantitative real-time PCR to detect fB mRNA expression

RNA isolation, cDNA synthesis, reverse transcription, and real-time RT-PCR were performed as described previously [87]. Briefly, RNA was isolated from heart tissues of fB^-/-^ and WT mice using Trizol (Life Technologies; CA). cDNA was reverse-transcribed using the High Capacity cDNA Reverse Transcription Kit (Applied Biosystems, NJ) as per the manufacturers’ instructions. Q-PCR was performed with TaqMan primers (Applied Biosystems) and run on the CFX96 Real-Time System (Bio-Rad Laboratories, CA). PCR products were normalized to the 18S control gene and expressed as fold increase over the mean value of fB^-/-^ heart samples using the ΔΔCt method.

fB primers are obtained as TaqMan probes from Applied Biosystems (Waltham, MA): Mm00433918_g1 and 18s primer: Mm03928990_g1.

### Immunohistochemical analysis of complement C3 deposition

Frozen sections were cut from the heart slices described in Section 2, fixed in acetone and stained with an FITC-labeled anti-C3c antibody (Dako, CA). Each section was imaged (2x objective lens) using channel 4 (for all fluorescence) to give total area. The C3 positive area was imaged (10x objective lens) and quantified by Image J software. The percentage of the total area that was C3 positive was determined.

### Patient enrollment, perioperative management and blood sampling

The prospective clinical study was approved by the Institutional Review Boards of SUNY Downstate Medical Center and Yale University School of Medicine (Approval#07–106). Adult patients (total 105), undergoing elective cardiac surgery with cardiopulmonary bypass (CPB), were enrolled in the study following their consent. All participants provided their written informed consents to participate in this study.

At the discretion of the attending cardiac surgeons, oral antiplatelet agents were discontinued within 7–10 days before surgery. Patients chronically treated with beta-blocking agents or statins continued these medications until the morning of surgery. Midazolam (1-2mg) was given as soon as the standard monitors (*i*.*e*., five-lead electrocardiogram with computerized analysis of repolarization, end tidal CO_2_ monitor and pulse oximetry) were applied and prior to insertion of the arterial line. Other monitors, including central venous pressure and pulmonary artery occlusion pressure were applied after induction of anesthesia. A smooth induction, aiming to maintain hemodynamic parameters as close as possible to each patient’s baseline, was used. Antifibrinolytic therapy with tranexamic acid (a 10-20mg/kg bolus followed by an infusion of 1-2mg/kg/hr.) was administered. CPB was carried out under hypothermia at approximately 32°C with a pump flow of 2.0–2.4 L/min/m^2^. The cardioplegic solution was delivered after clamping of the aorta. The hematocrit was maintained at ≥16% while patients were on bypass and >23% postoperatively. General anesthesia was maintained with a combination of opioid-volatile techniques and the depth of anesthesia was titrated to meet the requirements of the varying intensities of surgical stimulation. Boluses of vasoactive agents were given intraoperatively as necessary to maintain mean arterial pressure between 50 and 80 mm Hg. If, after aortic unclamping, sinus rhythm did not resume spontaneously, the heart was defibrillated. After termination of CPB, catecholamines were used at the discretion of the anesthesiologist. Postoperatively, patients were admitted to the CT-ICU under the care of the anesthesiologist and cardiac surgeon on duty.

Coronary sinus blood samples were collected after CPB prior to AXCL and 5 minutes after AXCL termination. Blood samples were centrifuged and the plasma stored at -80°C.

Of the 105 patients who consented to participate, complete sets of coronary sinus blood samples were collected from 56. Data from the remaining patients, where at least 1 blood collection was missed, were not included. Collections were missed due to the exigencies of cardiac surgery which prevent surgeons from taking time to sample blood for research. Relevant demographic and clinical parameters were collected (Table 1). Levels of cTnI were determined by ELISA (Calbiotech, CA) on plasma from peripheral blood samples collected pre-surgery and immediately post-surgery.

**Table 1:** Demographics and baseline data for the 56 patients in the study who underwent open heart surgery.

Age (years)	64 ± 13
Male	66%
Body mass index (kg/m^2^)	28 ± 5
Left ventricle ejection fraction (EF)	47 ± 18%
Diabetes mellitus	44%
Hypertension	94%
Hyperlipidemia	77%
Current smokers	25%
Type of cardiac procedure:	
Coronary artery bypass grafting (CABG)	36%
Valvular replacement	39%
Combined CABG and valvular replacement	25%
CPB time (min)	110 ± 40
AXCL (min)	72 ± 34

### ELISA measurement of patients’ Bb

Patients’ plasma levels of Bb were measured using the ELISA kit from Quidel (San Diego, CA) which detects the Bb component of activated fB but not native fB, nor the other fragment of fB activation, Ba.

### Statistical analysis

Statistical analyses were performed using IBM SPSS Software version 20 (IBM Corp., NY). For animal studies, an independent t-test with two tails and unequal variances was used to determine the statistical significance between the results for fB^-/-^ and WT mice. Descriptive data were summarized as the mean ± standard error of mean. For clinical studies, the patients’ demographic and relevant clinical data, together with plasma Bb levels, were entered into a Microsoft Excel database. Descriptive data were summarized as the mean ± standard deviation. A paired t-test with two tails and unequal variances was used to determine statistically significant differences in the levels of Bb and fB (from Western blotting) in coronary blood taken before and after AXCL. *P*ost hoc power analyses performed using G*Power 3.1 [86] showed >99% power for detection of differences in the levels of Bb in coronary blood taken before and after AXCL. Statistically significant correlations between the post-AXLC levels of Bb, the post-surgery levels of cTnI and AXCL time were determined using Spearman’s correlation. Box-charts were plotted using SigmaPlot 11 software (Systat Software, CA).

## Results

### Factor B deficiency limits cardiac I/R-induced myocardial necrosis in mice

**Fig 1:**
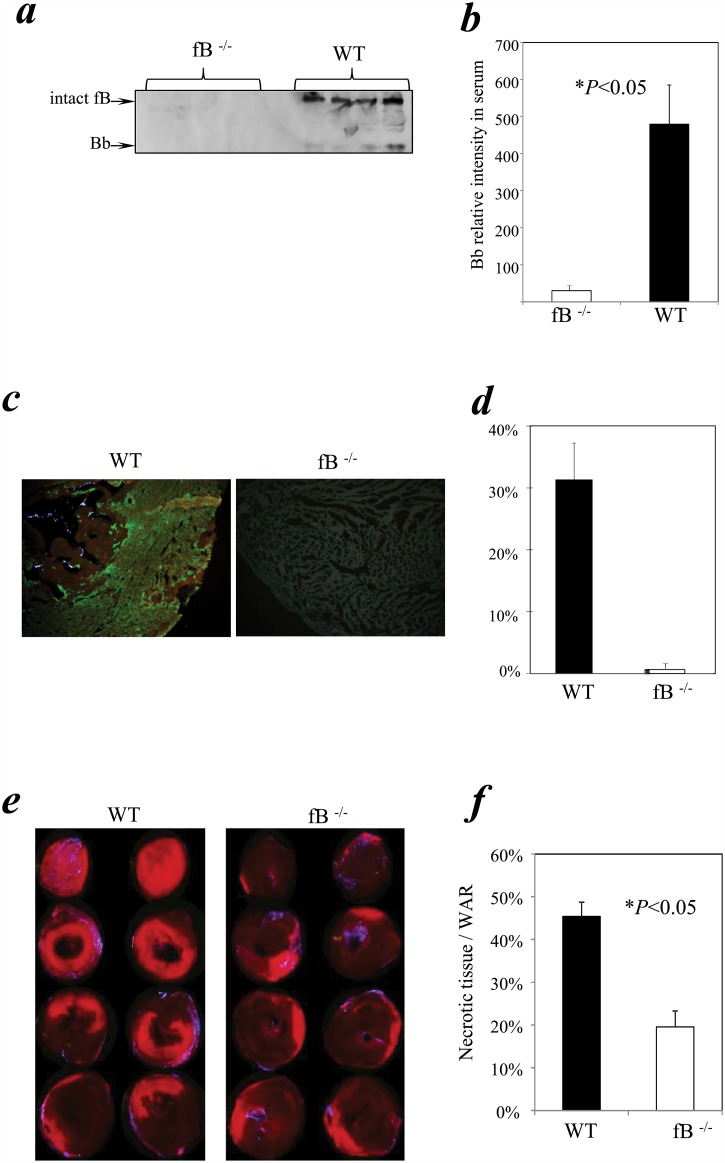
Factor B knockout mice experienced reduced myocardial necrosis and complement C3 deposition. fB^-/-^ mice and WT were used in a myocardial I/R model. The left anterior descending (LAD) coronary artery was occluded for 1 hr then reperfused for 24 hrs. Propidium iodide and blue fluorescent microspheres (BFM) (the latter after re-occlusion of the LAD) were injected *in vivo* just prior to heart harvesting to delineate the necrotic tissue and the tissue lacking circulation and therefore at risk for necrosis, respectively. ***(a)*** Circulating fB in the blood was significantly activated in WT mice (n = 4) but not in fB^-/-^ mice (n = 4). Serum obtained from cardiac puncture at the end of reperfusion was analyzed by Western blotting as described in Materials and Methods, Section 3. Each lane in the blot represented a separate mouse. Arrows indicate fB and Bb fragments. ***(b)*** The bar chart summarizes the relative intensities of Bb fragments. Bars represent Means ± SEM (* indicates *P*<0.05 compared with WT controls). ***(c)*** Cryosections prepared from the heart slices were stained with a FITC-tagged anti-C3 antibody. ***(d)*** Bar graph: bars indicate the percentage of total area that is C3 positive. ***(e)*** Necrotic tissue (bright red fluorescence) was visualized under a fluorescent microscope immediately after the harvest of hearts, using a 2x objective lens, in slices obtained by dividing the heart into four (top and bottom of each slice are adjacent in the figure). Non-ischemic tissue was defined by the blue fluorescence of BFM, the non-fluorescing tissue constituting weight of tissue at risk (WAR) (n = 7 per group). ***(f)*** Bar graph: Necrotic area expressed as % WAR as defined in the Materials and Methods, Section 2.

To test for a relationship between myocardial I/R injury and activation of the alternative pathway complement, we subjected fB^-/-^ and congenic WT control mice to 1 hour of surgically-induced myocardial ischemia (LAD coronary ligation) followed by 24 h of reperfusion. Assays performed on blood samples from WT mice showed activation of fB (manifested as detection of the activation product Bb) at 24 h, while no fB or Bb was detected in peripheral blood of fB^-/-^ mice (*P*<0.05, Fig 1a and 1b; S1 Fig; Part A in S1 File). When we examined the heart tissue for deposition of C3 activation products by immunohistochemistry, we observed that the absence of fB fully prevented C3 deposition (C3-positive staining area as a % of total section area: fB^-/-^ mice, 0 ± 0%; WT mice, 31 ± 6%, *P*<0.05; Fig 1c and 1d; Part B in S1 File). Remarkably, we also observed significantly less myocardial necrosis in the fB^-/-^ mice compared with the WT littermate controls (n = 7/per group; % ventricular tissue which was necrotic: 20 ± 4% versus 45 ± 3%, respectively, *P* < 0.05; Fig 1e and 1f; S2 Fig; Part C in S1 File). Together with the published literature [73, 77], the data indicates a central role for fB and the alternative pathway in the pathophysiology of myocardial I/R injury.

### Factor B from circulation contributes to myocardial necrosis in cardiac I/R

**Fig 2:**
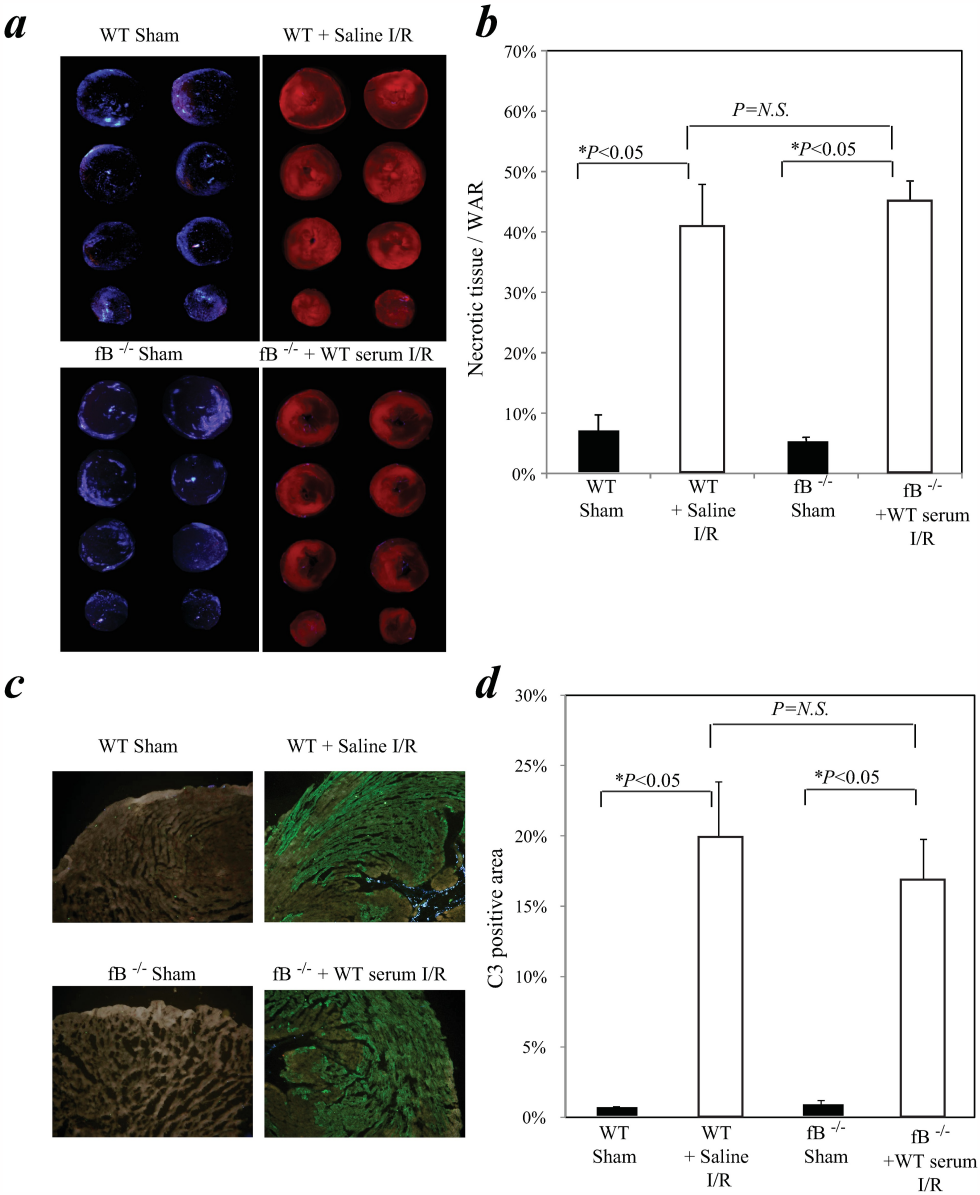
Factor B from circulation contributed myocardial necrosis in cardiac I/R. fB^-/-^ mice (n = 4) were re-constituted *i*.*v*. with 500ul WT serum (first 250ul i.v. 1 hour prior to surgery; second 250 μl i.v. 40 minutes prior to surgery) to provide an extracellular source of fB. WT mice (n = 4; similarly injected) were used as positive controls. Animals were subjected to myocardial IR (1 h ischemia/24 h reperfusion). Sham operated fB^-/-^ (n = 5) or WT mice (n = 5) were included as negative controls. ***(a)*** Myocardial necrosis was determined as done in Fig 1. ***(b)*** Bar graph: Necrotic area expressed as % WAR as defined in Fig 1. ***(c)*** Heart cryosections were stained with a FITC-tagged anti-C3 antibody. ***(d)*** Bar graph: bars indicate the percentage of total area that is C3 positive.

To determine the source of the fB which contributed to post-I/R myocardial necrosis, fB^-/-^ mice (n = 4) were re-constituted *i*.*v*. with WT serum to provide an extracellular source of fB (i.e. no local production) and subjected to myocardial IR as above (1 h ischemia / 24 h reperfusion). WT mice (similarly subjected to myocardial I/R) acted as positive controls (n = 4/group) and sham operated fB^-/-^ and WT mice served as negative controls. Reconstitution of fB^-/-^ mice with WT serum restored the myocardial necrosis (Fig 2a and 2b; S3 Fig; Part D in S1 File) and activated C3 deposition in the myocardium (Fig 2c and 2d; Part E in S1 File) after cardiac I/R.

**Fig 3:**
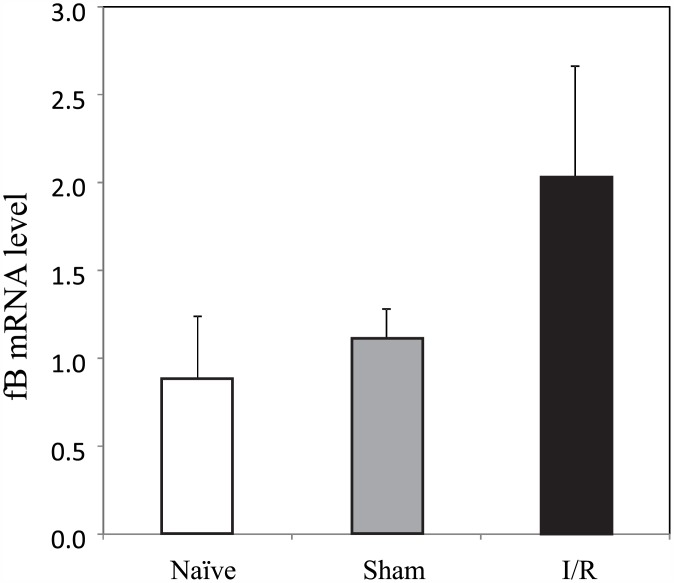
mRNA expression of fB in the WT hearts after IR. RNA were isolated from WT hearts without surgery (naïve group; n = 3), or sham operation (n = 10), or I/R operation (n = 12). cDNA were synthesized, reverse transcribed, and real-time RT-PCR were performed as described in Method Section.

To test whether I/R altered gene expression of fB in WT hearts, we measured myocardial fB mRNA levels by qPCR. These assays showed <2-fold differences (not statistically or physiologically different) between fB levels in hearts from naïve mice vs those that underwent I/R or sham surgeries (Fig 3; Part F in S1 File), further supporting the hypothesis that systemic rather than local derived fB is the key mediator of these effects.

### Activation of fB in patients’ coronary circulation during aortic cross-clamping in cardiac surgery

**Fig 4:**
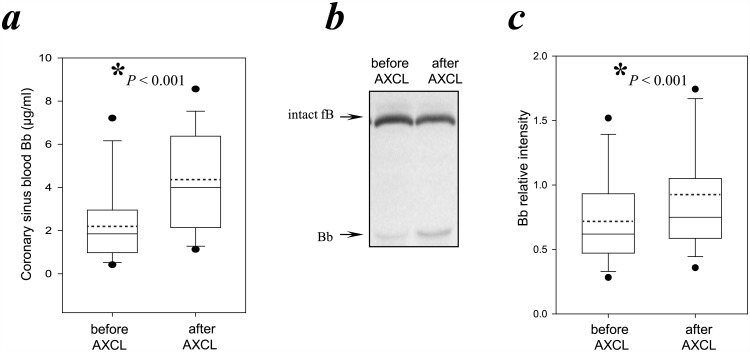
Activation of fB in the coronary circulation after AXCL. ***(a)*** Bb levels increased in the coronary circulation after the AXCL of human cardiac surgery. Coronary sinus blood was collected prior to AXCL and 5 minutes after AXCL termination. Bb levels were determined by ELISA using an antibody which detects the Bb component of activated fB but not native fB, nor the other fragment of fB activation, Ba. Statistical significances were analyzed as described in Materials and Methods, Section 8. Box-charts were plotted using SigmaPlot software. The boundary of the box closest to zero indicates the 25th percentile, while the boundary of the box farthest from zero indicates the 75th percentile. Error bars above and below the box indicate the 90th and 10th percentiles. The two filled circles above and below the box indicate the 95th and 5th percentiles. The solid line within the box marks the median, and the dotted line marks the mean (average). N = 56; * indicates statistical significance (*P* < 0.05). ***(b)*** Plasma from the coronary sinus blood obtained before the start of AXCL and 5 minutes after AXCL cessation was analyzed by Western blotting using a polyclonal antibody that detects fB and Bb. A representative blot is depicted showing fB positive bands from a patient’s plasma. Arrows indicate fB and Bb fragments. Band intensities on scanned images of such blots were quantified and normalized to the 93 kDa fB band of a control plasma and expressed as relative intensity. ***(c)*** A bar chart summarizes the relative intensities of Bb fragments.

To determine whether fB is activated by I/R in humans during cardiac surgery, we analyzed the Bb levels in plasma from coronary sinus blood collected prior to aortic cross clamping (AXCL) and immediately after its cessation. Consistent with the findings in mice, global heart ischemia (AXCL) and reperfusion (cessation of AXCL) was associated with higher coronary sinus blood Bb levels compared with those prior to application of AXCL ([Bb] prior to AXCL = 2.4 ± 2.0 μg/ml; [Bb] after AXCL cessation = 4.4 ± 2.9 μg/ml, *P* < 0.01) (Fig 4a; S4 Fig). Likewise, peripheral blood levels of Bb rose post-surgically ([Bb] prior to AXCL = 2.4 ± 1.8 μg/ml; [Bb] after AXCL = 5.0 ± 3.8 μg/ml, *P*<0.05. Data not shown). The pre-AXCL levels of Bb were similar between the coronary and peripheral blood but after reperfusion the Bb levels in the peripheral blood were slightly higher (*P* < 0.05) than in the coronary blood. The clinical relevance of this difference is unclear.

To determine whether these increases in Bb were caused by an increase in the production of the precursor, fB, and/or an increase in the activation rate of existing fB, we next performed Western blotting to separate and quantify the intact fB (93 kDa) and the cleaved product, active Bb (60 kDa) in coronary sinus blood samples. We reasoned that if fB production were increased during the ischemia caused by cross-clamping, then an increase in the amounts of both the intact fB and the Bb fragment would be expected in the post-AXCL samples. If fB activation to produce Bb used pre-existing fB, then an increase in the amount of the Bb fragment, but not the parent protein, would be expected.

The 60 kDa Bb band showed a significant 20% increase (Fig 4b and 4c) in intensity after AXCL (60 kDa band intensities relative to those of the normal control 93 kDa fB band: pre-AXCL = 0.74 ± 0.39; post-AXCL = 0.89 ± 0.46, *P* < 0.01). In addition, there was a small non-statistically significant decrease (3%) in the intensity of the 93 kDa fB band after cessation of AXCL (93 kB band intensities relative to those of the normal control 93 kDa band: pre-AXCL = 0.70 ± 0.19; post-AXCL = 0.68 ± 0.19, *P* = 0.201). Together, these results are consistent with the hypothesis that the increased levels of Bb after AXCL cessation are derived from activation of pre-existing fB.

### Activated fB levels correlate significantly with the postoperative increase in myocardial necrosis marker cTnI

**Fig 5:**
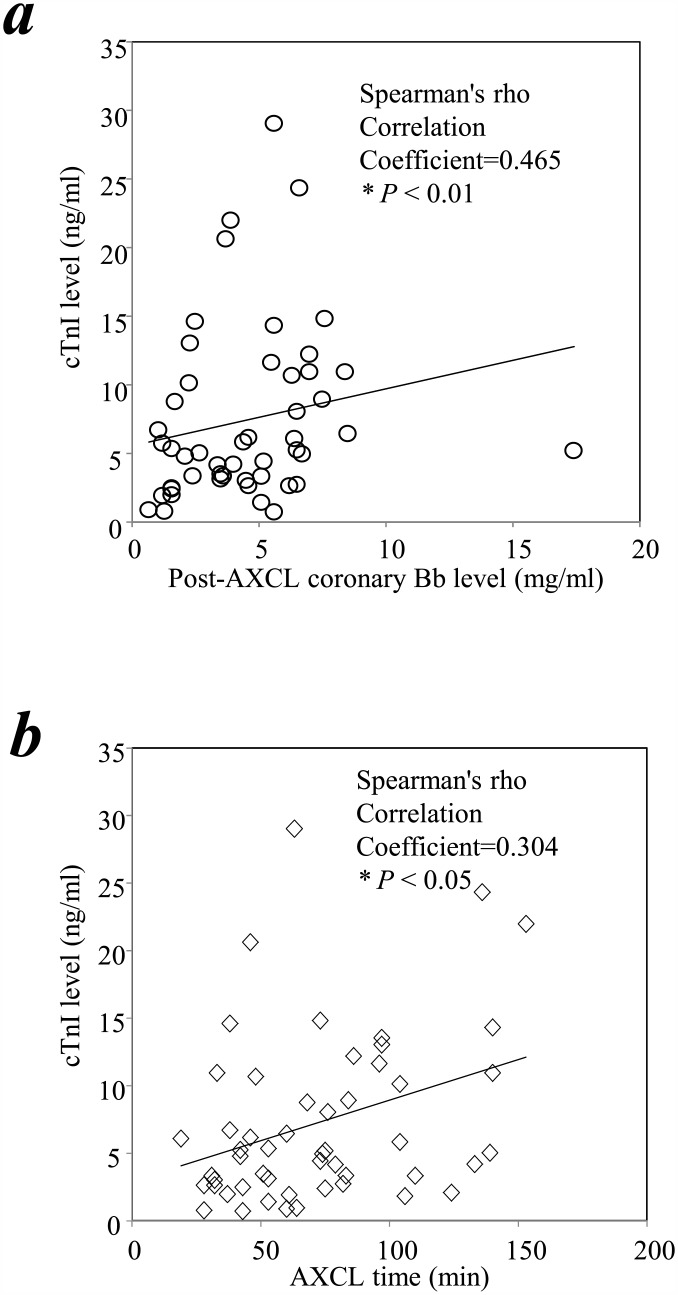
*(a)* The increases in coronary blood Bb levels immediately after AXCL correlated significantly with postoperative increases in cTn1 levels. ***(b)*** The increases in postoperative increases in cTn1 levels correlated significantly with AXCL time. Univariate analyses were carried out as described in Method. * indicates statistical significance.

To investigate the clinical significance of fB activation in the coronary circulation, we determined whether activated fB correlated with post-surgical levels of the myocardial necrosis marker cTnI. Peripheral blood cTnI levels increased significantly following cardiac surgery compared with the immediate pre-surgery values (Table 2), consistent with previous literature [7, 8, 81]. A univariate analysis showed that coronary Bb levels immediately after release of AXCL directly correlated with post-surgical peripheral blood cTnI levels (Spearman’s rho correlation coefficient = 0.465, *P* < 0.01; Fig 5a). Peripheral blood cTnI levels correlated with AXCL time (Spearman’s rho correlation coefficient = 0.304, *P* < 0.05; Fig 5b) but Bb levels did not (*P* >0.05). To assess whether the AXCL time influenced the relationship between Bb and cTn1 we repeated the analyses using the mean and median ACXL times as cutoffs. These analyses showed that Bb levels correlated with cTn1 above and below the tested thresholds (not shown).

**Table 2:** Levels of the myocardial necrosis marker cTnI in the peripheral blood increased significantly following cardiac surgery.

Time points	cTnI level (ng/ml)	*p*-value
1. immediately pre-surgery	0.89 ± 0.44	
2. immediately post-surgery	7.03 ± 0.87	<0.01*

* indicates statistical significance between cTnI levels at post-surgery and pre-surgery levels.

## Discussion

Building upon the emerging knowledge regarding mechanisms underlying acute myocardial I/R injury (reviewed in-depth elsewhere [18, 88–90]), various strategies aimed at preventing myocardial I/R injury since the 1980s have shown promise in animal models and small human trials [16–22]. Nonetheless, to date, none of the large trials targeting various mechanisms thought to be involved in I/R injury have shown benefit in reducing post-MI damage [18, 23–25, 29–31]. New mechanistic insights are required to drive novel therapeutic approaches.

Our new findings demonstrate that activation of circulating systemic fB is central to myocardial damage in a murine model of surgically-induced cardiac I/R. *Factor B*^*-/-*^ mice showed a significant reduction in complement C3 deposition and a remarkable abrogation of myocardial necrosis compared to WT littermates (Fig 1). Reconstitution of *fB*^*-/-*^ mice with WT serum restored both C3 deposition and myocardial damage (Fig 2). These results add to previous studies describing the critical role of the complement alternative pathway in other models of cardiac ischemic injury (e.g. permanent occlusion of coronary arteries, after heart transplantation) [91–94], and on late post-ischemic organ sequelae such as cardiac hypertrophy [95]. The alternative pathway has also been shown to be important in renal IR injury [96–100] but not gastrointestinal IR damage [101, 102] suggesting that there are important organ specific mechanistic differences that need to be explored further.

The current paradigm of complement activation after myocardial I/R injury developed from prior work by our lab, among many others, is that ischemia of cardiac cells results in exposure of neo-antigens on the cell surface that can be recognized upon reperfusion by naturally occurring circulating IgM antibodies[77, 78, 103, 104]. The lectin pathway can recognize these antibody-neoantigen complexes and appears to be the dominant pathway of C3 activation [73, 81, 105]. C3 activation is then amplified in a fB-alternative pathway-dependent manner. The exact mechanism by which fB amplifies complement activity after cardiac I/R injury (the standard model of C3 hydrolysis[106] [107–110], the properidin-directed model[111, 112, 113, 114, 115], or the IgG-mediated model[116, 117]) needs further study.

The downstream effects of complement activation after ischemia reperfusion injury has been well described and include production of the anaphylatoxins C3a/C5a as well as the membrane attack complex C5b-9 [118, 119]. Multiple experimental models have already shown that targeting these mechanisms effectively reduces tissue damage (reviewed in depth elsewhere) [118, 119], but large randomized trials using blockade of C5 did not alter the incidence or severity of I/R injury [120, 121]. Our new findings show that blocking the upstream complement amplification loop is sufficient to mitigate I/R-injury. Whether and how factor B deficiency affects the distal effector mechanisms of the complement cascade still needs to be formally tested.

Complement activation has been implicated in human I/R injury [122–128], but not without some controversy [129–133]. In clinical trials, blockade of the common terminal complement pathway using the anti-complement C5 drug Pexelizumab failed to provide statistically significant improvements in outcomes for cardiac patients undergoing coronary artery bypass graft surgery [134] or PCI [120]. It is possible that this lack of efficacy relates to the fact that targeting downstream C5 cleavage does not affect production of upstream alternative pathway-amplified complement effector molecules including C3-derived C3a and C3b. Two previous clinical studies of patients post cardiopulmonary bypass showed acute increases in systemic serum activated fB [135, 136] as early as 30mins and 1hr post-procedure and concomitant elevations in C3 and C4 activation products, which are all proximal to complement C5. Building upon these findings, we show for the first time that fB activation, as determined by Bb levels, is also increased in samples drawn directly from the coronary circulation immediately following cessation of global heart ischemia induced by ACXL (Fig 4a) supporting the hypothesis that the systemic activation of the complement alternative pathway originates in part from the reperfused heart. Whether fB participates in the pathogenesis of cardiac necrosis in humans with acute myocardial infarction and whether targeting fB will improve outcomes following acute myocardial infarction remain to be determined through future trials.

We also provide evidence that this early activated fB is derived predominantly from pre-existing fB rather than large de novo fB generation. Western blot analysis showed a rise in Bb and a trend toward less fB in serum isolated from the coronary sinus after AXCL leading to a relative rise in Bb vs fB levels (Fig 4b and 4c). Further, as noted above, the myocardial necrosis and complement deposition that was seen in hearts of WT mice after I/R was able to be fully restored in fB^-/-^ mice after reconstitution with WT serum alone (Fig 2c and 2d). Correspondingly we did not detect increased fB mRNA in WT hearts experiencing I/R relative to sham surgery or naïve mice (Fig 3) supporting the argument that systemic fB is the dominant source involved in the injury.

We also show that the Bb levels in the coronary circulation correlated with immediate postoperative levels of the myocardial necrosis marker cTnI (Fig 5) which has been shown to inversely correlate with clinical outcomes [7, 8, 137, 138]. Given our murine findings suggesting the central role of alternative complement pathway activation and myocardial injury after I/R, it is intriguing to consider that activated fB may serve not only as a predictive biomarker but also a potential therapeutic target. Still, since the postoperative cTnI level correlates with length of AXCL, it is possible that the association between intracoronary fB activation (Bb levels) and cardiac necrosis (cTnI levels) reflects the no-flow period rather than a causative relationship. Further, peripheral levels of Bb rose post-surgically and were statistically significantly higher than coronary sinus samples. This could be due to additional Bb generation occurring distal to the collection point of the coronary sinus blood or may be related to a mechanism of complement activation occurring outside the reperfused organ that warrants further investigation.

In summary our new results from our animal and clinical observations provide the first evidence that fB contributes directly to the myocardial necrosis that occurs early after surgical cardiac I/R injury and provides the foundation for testing fB inhibitors to limit IR injury and improve patient outcomes.

## Supporting information

S1 Fig Additional experiments showing circulating fB in the blood was significantly activated in WT mice but not in fB^-/-^ mice. It is of note that Bb levels in WT mice (n = 5) did vary from mouse to mouse, and the highest level was about 5 times more than the lowest one. The lowest Bb level in WT mice was indistinguishable from that of fB^-/-^ mice (n = 4) by the current Western blot method using an anti-human fB antibody and a donkey anti-goat antibody conjugated with HRP (Rockland, PA) (developed with an ECL Western blotting kit from Thermo Scientific, NJ).
(EPS)

S2 Fig Enlarged view of the heart sections from WT mice injected with saline and undergone heart I/R. White arrows indicate BFM.
(EPS)

S3 Fig Enlarged view of the heart sections from fB ^-/-^ mice injected with WT serum and undergone heart I/R. White arrows indicate BFM.
(EPS)

S4 Fig Activation of fB in the coronary circulation after AXCL. Plasma from the coronary sinus blood obtained before the start of AXCL and 5 minutes after AXCL cessation was analyzed by Western blotting using a polyclonal antibody that detects fB and Bb. Each diamond and circle represents one patient. * indicates statistical significance between the groups (*P* < 0.05).
(EPS)

S1 File Part A: Original data set of Fig 1b; Part B: Original data set of Fig 1d. Part C: Original data set of Fig 1f; Part D: Original data set of Fig 2b; Part E: Original data set of Fig 2d; Part F: Original data set of Fig 3.
(XLSX)
